# The 4-(3-chloro-4-methyl­phen­yl)-1,2,3,5-dithia­diazol-3-yl radical

**DOI:** 10.1107/S1600536811034751

**Published:** 2011-08-27

**Authors:** Jacqueline M. Cole, Christine M. Aherne, Judith A. K. Howard, Arthur J. Banister, Paul G. Waddell

**Affiliations:** a Cavendish Laboratory, University of Cambridge, J. J. Thomson Avenue, Cambridge CB3 0HE, England; b Department of Chemistry, University of Durham, South Road, Durham, DH1 3LE, England

## Abstract

The asymmetric unit of the title compound, C_8_H_6_ClN_2_S_2_, comprises two mol­ecules forming a dimer *via* π–π stacking inter­actions [centroid–centroid distance = 3.634 (10) Å] and intra­dimer S⋯S contacts [3.012 (4) and 3.158 (4) Å] between the two mol­ecules in a *cis*-antarafacial arrangement.

## Related literature

For the properties of the 4-methyl­phenyl dithia­diazolyl radical, see: Boeré *et al.* (1992 ▶). For similar phenyl dithia­diazolyl radical structures, see: Allen *et al.* (2009 ▶); Clarke *et al.* (2010 ▶). For notes on the configurations adopted by phenyl dithia­diazolyl radicals in their crystal structures, see: Aherne *et al.* (1993 ▶). 
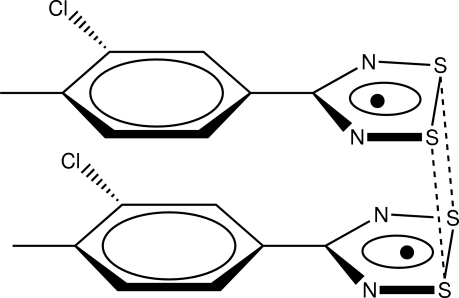


## Experimental

### 

#### Crystal data

C_8_H_6_ClN_2_S_2_
*M* *_r_* = 229.72
Monoclinic, 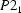

*a* = 5.937 (3) Å
*b* = 13.407 (3) Å
*c* = 11.573 (3) Å
β = 95.87 (4)°
*V* = 916.3 (6) Å^3^
*Z* = 4
Mo *K*α radiation
μ = 0.82 mm^−1^
*T* = 150 K
0.25 × 0.18 × 0.15 mm

#### Data collection

Rigaku AFC-6S diffractometer
1655 measured reflections
1499 independent reflections
1054 reflections with *I* > 2σ(*I*)
*R*_int_ = 0.103
θ_max_ = 24.0°
3 standard reflections every 200 reflections  intensity decay: none

#### Refinement

*R*[*F* ^2^ > 2σ(*F* ^2^)] = 0.042
*wR*(*F* ^2^) = 0.098
*S* = 1.09
1499 reflections
237 parameters
1 restraint
H-atom parameters constrained
Δρ_max_ = 0.44 e Å^−3^
Δρ_min_ = −0.38 e Å^−3^
Absolute structure: Flack (1983 ▶), 169 Friedel pairs
Flack parameter: −0.16 (19)

### 

Data collection: *MSC/AFC Diffractometer Control Software* (Molecular Structure Corporation, 1991 ▶); cell refinement: *MSC/AFC Diffractometer Control Software*; data reduction: *TEXSAN* (Molec­ular Structure Corporation, 1989 ▶); program(s) used to solve structure: *SHELXS97* (Sheldrick, 2008 ▶); program(s) used to refine structure: *SHELXL97* (Sheldrick, 2008 ▶); molecular graphics: *SHELXTL* (Sheldrick, 2008 ▶); software used to prepare material for publication: *WinGX* publication routines (Farrugia, 1999 ▶).

## Supplementary Material

Crystal structure: contains datablock(s) global, I. DOI: 10.1107/S1600536811034751/bt5594sup1.cif

Structure factors: contains datablock(s) I. DOI: 10.1107/S1600536811034751/bt5594Isup2.hkl

Additional supplementary materials:  crystallographic information; 3D view; checkCIF report

## Figures and Tables

**Fig. 1.**
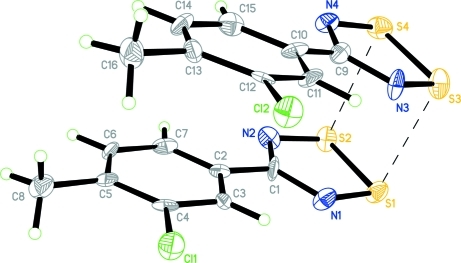
The structure of the title compound with displacement ellipsoids drawn at the 50% probability level showing S···S contacts (dashed lines).

## supplementary crystallographic information

### Comment

As observed in similar structures (Aherne *et al.* 1993; Allen *et al.* 2009 and Clarke *et al.* 2010), within the planar CS_2_N_2_ rings the C—N and S—N bonds distances exhibit intermediate values between those of standard single and double bonds indicating the delocalized nature of the radical about the N—C—N fragment. Though the S—S distance is unusually long it is comparable to similar phenyl dithiadiazolyl radical structures. Intradimer contacts include π-π stacking interactions between the aryl rings of the 3-chloro-4-methylphenyl dithiadiazolyl radicals with a centroid-to-centroid distance of 3.634 (10) Å, and two *cis*-antarafacial S···S contacts (S1···S3 = 3.012 (4) Å; S2···S4 = 3.158 (4) Å).

### Experimental

Lithium hexamethyldisilazane (1.67 g, 0.01 mol) was added to 3-chloro-4-methylbenzonitrile (1.51 g, 0.01 mol) in ethanol (40 ml) and stirred for 3 h. To the resulting yellow solution SCl_2_ (1.27 ml, 0.02 mol) and diethyl ether (25 ml) was added producing a red solution containing the 3-chloro-4-methylphenyl dithiadiazolyl cation (yield: 91°). The radical was formed upon reduction of the cation (1.43 g, 5 mmol) with a Zn(Cu) couple (0.18 g, 2.8 mmol) as the reducing agent in THF (25 ml). Crystals suitable for X-ray crystallography were grown *via* sublimation of the product under vacuum at 373 K.

### Refinement

H atoms were positioned geometrically and refined as riding on their parent atoms, with C—H = 0.930 Å and *Uiso(H)* = 1.2*Ueq(C)*. Methyl hydrogen atoms were modeled in a similar fashion C—H = 0.960 Å and *Uiso(H)* = 1.5*Ueq(C)*. The most disagreeable reflections were omitted; six reflections exhibiting a Δ(F^2^) value greater than 5 su were removed. The absolute structure was determined with a Flack (1983) parameter of -0.16 (19), using 169 reflections.

### Crystal data

**Table:**

C_8_H_6_ClN_2_S_2_	*F*(000) = 468
*M**_r_* = 229.72	*D*_x_ = 1.665 Mg m^−^^3^
Monoclinic, *P*2_1_	Mo *K*α radiation, λ = 0.71073 Å
Hall symbol: P 2yb	Cell parameters from 20 reflections
*a* = 5.937 (3) Å	θ = 3.0–11.0°
*b* = 13.407 (3) Å	µ = 0.82 mm^−^^1^
*c* = 11.573 (3) Å	*T* = 150 K
β = 95.87 (4)°	Prism, colourless
*V* = 916.3 (6) Å^3^	0.25 × 0.18 × 0.15 mm
*Z* = 4	

### Data collection

**Table:**

Rigaku AFC-6S diffractometer	θ_max_ = 24.0°, θ_min_ = 3.5°
graphite	*h* = 0→6
ω scans	*k* = 0→15
1655 measured reflections	*l* = −13→13
1499 independent reflections	3 standard reflections every 200 reflections
1054 reflections with *I* > 2σ(*I*)	intensity decay: none
*R*_int_ = 0.103	

### Refinement

**Table:**

Refinement on *F*^2^	Secondary atom site location: difference Fourier map
Least-squares matrix: full	Hydrogen site location: inferred from neighbouring sites
*R*[*F*^2^ > 2σ(*F*^2^)] = 0.042	H-atom parameters constrained
*wR*(*F*^2^) = 0.098	*w* = 1/[σ^2^(*F*_o_^2^) + (0.0132*P*)^2^ + 2.2783*P*] where *P* = (*F*_o_^2^ + 2*F*_c_^2^)/3
*S* = 1.09	(Δ/σ)_max_ < 0.001
1499 reflections	Δρ_max_ = 0.44 e Å^−^^3^
237 parameters	Δρ_min_ = −0.38 e Å^−^^3^
1 restraint	Absolute structure: Flack (1983), 169 Friedel pairs
Primary atom site location: structure-invariant direct methods	Flack parameter: −0.16 (19)

### Special details

**Table:**

Geometry. All s.u.'s (except the s.u. in the dihedral angle between two l.s. planes) are estimated using the full covariance matrix. The cell s.u.'s are taken into account individually in the estimation of s.u.'s in distances, angles and torsion angles; correlations between s.u.'s in cell parameters are only used when they are defined by crystal symmetry. An approximate (isotropic) treatment of cell s.u.'s is used for estimating s.u.'s involving l.s. planes.
Refinement. Refinement of *F*^2^ against ALL reflections. The weighted *R*-factor *wR* and goodness of fit *S* are based on *F*^2^, conventional *R*-factors *R* are based on *F*, with *F* set to zero for negative *F*^2^. The threshold expression of *F*^2^ > 2σ(*F*^2^) is used only for calculating *R*-factors(gt) *etc*. and is not relevant to the choice of reflections for refinement. *R*-factors based on *F*^2^ are statistically about twice as large as those based on *F*, and *R*- factors based on ALL data will be even larger.

### Fractional atomic coordinates and isotropic or equivalent isotropic displacement parameters (Å^2^)

**Table:**

	*x*	*y*	*z*	*U*_iso_*/*U*_eq_	
S2	0.5244 (5)	0.3269 (2)	−0.1014 (2)	0.0271 (7)	
S1	0.2324 (4)	0.3359 (2)	−0.0142 (2)	0.0263 (7)	
N2	0.6580 (15)	0.4154 (7)	−0.0244 (7)	0.025 (2)	
N1	0.3323 (14)	0.4260 (6)	0.0724 (7)	0.024 (2)	
C1	0.5430 (17)	0.4558 (8)	0.0533 (9)	0.024 (2)	
C2	0.6477 (16)	0.5355 (8)	0.1287 (8)	0.017 (2)	
C6	0.9463 (14)	0.6549 (7)	0.1743 (7)	0.019 (2)	
H6	1.0823	0.6826	0.1567	0.023*	
C7	0.8533 (15)	0.5770 (8)	0.1066 (8)	0.021 (2)	
H7	0.9289	0.5524	0.046	0.025*	
C3	0.5398 (15)	0.5719 (7)	0.2224 (8)	0.020 (2)	
H3	0.4034	0.5447	0.2401	0.024*	
C5	0.8434 (15)	0.6933 (8)	0.2681 (8)	0.021 (2)	
C8	0.9427 (16)	0.7782 (8)	0.3392 (8)	0.026 (2)	
H8A	1.061	0.8085	0.3007	0.039*	
H8B	1.004	0.7542	0.414	0.039*	
H8C	0.827	0.8267	0.3488	0.039*	
C4	0.6413 (15)	0.6499 (7)	0.2885 (7)	0.018 (2)	
Cl1	0.5049 (4)	0.6884 (2)	0.4074 (2)	0.0278 (7)	
S3	0.3937 (5)	0.1726 (2)	0.1531 (2)	0.0302 (7)	
S4	0.7080 (4)	0.1669 (2)	0.0853 (2)	0.0293 (7)	
Cl2	0.5678 (4)	0.47502 (19)	0.6289 (2)	0.0259 (6)	
N4	0.8253 (12)	0.2511 (6)	0.1763 (7)	0.0208 (19)	
C10	0.7757 (15)	0.3556 (7)	0.3403 (7)	0.018 (2)	
C15	0.9878 (16)	0.4033 (8)	0.3377 (8)	0.025 (2)	
H15	1.079	0.3871	0.2797	0.031*	
C9	0.6891 (16)	0.2851 (8)	0.2539 (8)	0.021 (2)	
C12	0.7250 (15)	0.4511 (7)	0.5142 (7)	0.014 (2)	
C11	0.6480 (15)	0.3831 (7)	0.4301 (8)	0.022 (2)	
H11	0.506	0.3544	0.4332	0.027*	
C14	1.0611 (14)	0.4740 (8)	0.4206 (7)	0.023 (2)	
H14	1.2004	0.5047	0.4161	0.027*	
C13	0.9336 (16)	0.5008 (7)	0.5106 (8)	0.020 (2)	
C16	1.0139 (17)	0.5799 (8)	0.6005 (9)	0.029 (3)	
H16A	0.8912	0.6245	0.6114	0.043*	
H16B	1.1369	0.6169	0.5738	0.043*	
H16C	1.0639	0.5482	0.673	0.043*	
N3	0.4777 (14)	0.2515 (7)	0.2537 (7)	0.025 (2)	

### Atomic displacement parameters (Å^2^)

**Table:**

	*U*^11^	*U*^22^	*U*^33^	*U*^12^	*U*^13^	*U*^23^
S2	0.0299 (17)	0.0296 (16)	0.0226 (14)	−0.0059 (15)	0.0067 (12)	−0.0050 (12)
S1	0.0193 (14)	0.0277 (15)	0.0311 (15)	−0.0007 (13)	−0.0007 (12)	−0.0104 (14)
N2	0.031 (5)	0.025 (5)	0.022 (4)	0.001 (4)	0.012 (4)	0.001 (4)
N1	0.019 (5)	0.026 (5)	0.026 (5)	0.000 (4)	0.001 (4)	−0.011 (4)
C1	0.018 (5)	0.022 (6)	0.031 (6)	−0.013 (5)	−0.005 (4)	0.006 (5)
C2	0.015 (5)	0.019 (6)	0.018 (5)	0.007 (4)	0.002 (4)	0.001 (4)
C6	0.008 (4)	0.025 (7)	0.023 (5)	−0.002 (4)	−0.001 (4)	0.004 (4)
C7	0.022 (6)	0.027 (6)	0.014 (5)	0.007 (5)	−0.002 (4)	0.007 (5)
C3	0.015 (5)	0.012 (5)	0.032 (6)	0.001 (4)	−0.005 (5)	0.007 (5)
C5	0.015 (5)	0.018 (6)	0.030 (5)	0.003 (5)	−0.007 (4)	0.008 (5)
C8	0.022 (5)	0.031 (6)	0.024 (5)	−0.001 (5)	−0.002 (5)	−0.004 (5)
C4	0.016 (5)	0.026 (7)	0.012 (5)	0.013 (5)	0.000 (4)	−0.003 (5)
Cl1	0.0259 (13)	0.0279 (17)	0.0310 (14)	−0.0008 (13)	0.0090 (10)	−0.0078 (13)
S3	0.0353 (15)	0.0314 (17)	0.0242 (13)	−0.0117 (14)	0.0047 (11)	−0.0063 (13)
S4	0.0318 (15)	0.0270 (17)	0.0287 (14)	0.0049 (14)	0.0005 (12)	−0.0049 (13)
Cl2	0.0266 (13)	0.0286 (16)	0.0240 (13)	−0.0004 (14)	0.0094 (10)	−0.0073 (13)
N4	0.019 (4)	0.019 (5)	0.025 (5)	0.005 (4)	0.003 (4)	−0.011 (4)
C10	0.015 (5)	0.031 (7)	0.008 (4)	0.008 (5)	−0.003 (4)	0.006 (4)
C15	0.028 (6)	0.028 (6)	0.022 (5)	0.005 (5)	0.010 (4)	0.008 (5)
C9	0.019 (5)	0.027 (6)	0.017 (5)	−0.003 (5)	0.001 (4)	0.010 (5)
C12	0.018 (5)	0.012 (5)	0.012 (5)	0.009 (4)	0.000 (4)	0.002 (4)
C11	0.013 (5)	0.026 (6)	0.026 (5)	0.001 (5)	−0.008 (4)	0.005 (5)
C14	0.012 (5)	0.034 (6)	0.022 (5)	−0.004 (5)	0.001 (4)	0.002 (5)
C13	0.023 (6)	0.013 (5)	0.024 (6)	−0.003 (4)	−0.003 (4)	0.002 (4)
C16	0.029 (6)	0.021 (6)	0.037 (6)	−0.005 (5)	0.008 (5)	−0.012 (5)
N3	0.023 (5)	0.030 (5)	0.024 (4)	−0.013 (4)	0.011 (4)	−0.008 (4)

### Geometric parameters (Å, °)

**Table:**

S2—N2	1.639 (10)	S3—N3	1.613 (9)
S2—S1	2.096 (3)	S3—S4	2.098 (4)
S1—N1	1.641 (8)	S4—N4	1.648 (8)
N2—C1	1.302 (13)	Cl2—C12	1.728 (9)
N1—C1	1.352 (13)	N4—C9	1.348 (12)
C1—C2	1.476 (13)	C10—C11	1.398 (13)
C2—C7	1.388 (13)	C10—C15	1.415 (13)
C2—C3	1.403 (13)	C10—C9	1.432 (13)
C6—C7	1.387 (13)	C15—C14	1.387 (13)
C6—C5	1.397 (12)	C15—H15	0.93
C6—H6	0.93	C9—N3	1.333 (12)
C7—H7	0.93	C12—C11	1.377 (13)
C3—C4	1.395 (13)	C12—C13	1.411 (13)
C3—H3	0.93	C11—H11	0.93
C5—C4	1.375 (12)	C14—C13	1.396 (13)
C5—C8	1.491 (14)	C14—H14	0.93
C8—H8A	0.96	C13—C16	1.528 (13)
C8—H8B	0.96	C16—H16A	0.96
C8—H8C	0.96	C16—H16B	0.96
C4—Cl1	1.746 (8)	C16—H16C	0.96
			
N2—S2—S1	94.3 (3)	N3—S3—S4	94.2 (3)
N1—S1—S2	94.1 (3)	N4—S4—S3	94.0 (3)
C1—N2—S2	114.7 (7)	C9—N4—S4	114.5 (7)
C1—N1—S1	113.6 (7)	C11—C10—C15	116.6 (9)
N2—C1—N1	123.3 (10)	C11—C10—C9	120.6 (8)
N2—C1—C2	119.3 (9)	C15—C10—C9	122.7 (8)
N1—C1—C2	117.3 (9)	C14—C15—C10	120.7 (8)
C7—C2—C3	118.8 (9)	C14—C15—H15	119.6
C7—C2—C1	120.4 (9)	C10—C15—H15	119.6
C3—C2—C1	120.7 (9)	N3—C9—N4	120.9 (9)
C7—C6—C5	122.4 (9)	N3—C9—C10	119.7 (8)
C7—C6—H6	118.8	N4—C9—C10	119.4 (8)
C5—C6—H6	118.8	C11—C12—C13	121.4 (8)
C6—C7—C2	120.3 (9)	C11—C12—Cl2	119.9 (7)
C6—C7—H7	119.9	C13—C12—Cl2	118.7 (7)
C2—C7—H7	119.9	C12—C11—C10	122.3 (9)
C4—C3—C2	118.6 (9)	C12—C11—H11	118.9
C4—C3—H3	120.7	C10—C11—H11	118.9
C2—C3—H3	120.7	C15—C14—C13	122.4 (9)
C4—C5—C6	115.8 (9)	C15—C14—H14	118.8
C4—C5—C8	122.1 (9)	C13—C14—H14	118.8
C6—C5—C8	122.1 (9)	C14—C13—C12	116.5 (9)
C5—C8—H8A	109.5	C14—C13—C16	122.1 (9)
C5—C8—H8B	109.5	C12—C13—C16	121.4 (8)
H8A—C8—H8B	109.5	C13—C16—H16A	109.5
C5—C8—H8C	109.5	C13—C16—H16B	109.5
H8A—C8—H8C	109.5	H16A—C16—H16B	109.5
H8B—C8—H8C	109.5	C13—C16—H16C	109.5
C5—C4—C3	124.0 (8)	H16A—C16—H16C	109.5
C5—C4—Cl1	119.5 (7)	H16B—C16—H16C	109.5
C3—C4—Cl1	116.4 (7)	C9—N3—S3	116.4 (7)
